# Examining antecedents of parents’ privacy concerns and mindful sharenting behaviors: through a lens of protection motivation theory

**DOI:** 10.3389/fpsyg.2026.1782981

**Published:** 2026-08-13

**Authors:** Seounmi Youn, Zhao Peng

**Affiliations:** 1 Department of Marketing Communication, Emerson College, Boston, MA, United States; 2 Department of Journalism, Emerson College, Boston, MA, United States

**Keywords:** children’s age, mindful sharenting, privacy concerns, privacy protective behaviors, protection motivation theory, threat-coping appraisals

## Abstract

**Introduction:**

Awareness of the risks associated with sharing children’s information online motivates parents to practice mindful sharenting, a behavior characterized by the use of protective strategies during online sharenting. This study employs Protection Motivation Theory (PMT) as a theoretical framework to empirically investigate the factors that predict these behaviors.

**Methods:**

An cross-sectional online survey was conducted with 603 U.S. parents to test a structural equation model examining how threat appraisals, coping appraisals, and privacy concerns influence protective behaviors.

**Results:**

Indicate that among threat appraisal, perceived risk severity, susceptibility, and the extrinsic benefit of impression management positively relate to parents’ privacy concerns. Among coping appraisal, response costs positively relate to these concerns. Consequently, these privacy concerns are associated with both photo-related and general mindful sharenting behaviors.

**Discussion:**

The findings also reveal that a child’s age moderates these relationships: the links between privacy concerns and both mindful sharenting behaviors are more pronounced for parents with younger children (≤9 years) than parents of older children (>9 years). Practically, the study highlights the need for educational initiatives that upskill parents to manage privacy risks effectively.

## Introduction

Sharenting refers to the behavior in which parents, intentionally or inadvertently, disclose their children’s personal information to the public in mediated spaces (Peng, 2024). As sharenting involves disclosing children’s personal information without proper privacy protection, it could entail privacy risks for children, such as identity theft, cyberbullying, and digital kidnapping (Amon et al., 2022; Say et al., 2025). Recognizing the risks of sharing their children’s information online, parents start to employ protective strategies to safeguard their children’s identity and privacy, a behavior Walrave et al. (2023) termed “mindful sharenting.”

Mindful sharenting is the thought-out version of regular sharenting. Like regular sharenting, mindful sharenting can provide parents with benefits such as enjoyment, emotional support, affirmation of parenting competences, and impression management (Peng, 2024). However, unlike regular sharenting, which is primarily driven by social norms and benefits, mindful sharenting is driven by parents’ self-reflection and risk awareness (Walrave et al., 2023). Alarmed by negative personal experiences or anecdotes from their social circles, parents recognize that once information is shared, control over its flow and purpose is lost (Amon et al., 2022; Autenrieth, 2018). To enjoy the psychological and social benefits of sharenting while simultaneously protecting privacy, parents adopt specific strategies such as posting false identities, restrict audience access, or regulate sharenting behaviors of other family members (Lieberknecht et al., 2026; Walrave et al., 2022; Walrave et al., 2023).

Previous research has primarily focused on parents’ motivations, perceived consequences, factors influencing sharenting, children’s reactions, and the negotiation process between parents and children regarding sharenting (Briazu et al., 2021; Cino and Wartella, 2021; Esfandiari and Yao, 2022; Fox and Hoy, 2019; Hoy et al., 2023; Ouvrein and Verswijvel, 2019, 2021; Walrave et al., 2022). While studies have shown evolving attitudes towards sharenting and increased awareness of potential risks (e.g., Williams-Ceci et al., 2021; Say et al., 2025), there have been limited studies exploring psychological factors such as threat and coping appraisals that motivate parents to engage in their mindful sharenting behaviors. Walrave et al. (2023) laid the groundwork for understanding mindful sharenting and its underlying motives. However, their study is qualitative in nature and further quantitative research is required to empirically examine the intertwined relationship between threat-coping appraisals and mindful sharenting.

Scholars have examined motivations and consequences of sharenting with a lens of privacy calculus theory (Peng, 2024), communication privacy management theory (Siibak and Traks, 2019; Walrave et al., 2022; Walrave et al., 2023), and self-presentation theory (Kallioharju et al., 2023; Lazard et al., 2019; Williams-Ceci et al., 2021). While these studies have offered insights on sharenting behaviors, scholars call for research that applies PMT to mindful sharenting behaviors due to theoretical efficacy. For example, Lieberknecht et al. (2026) found that parents’ assessment of children’s vulnerability to privacy invasion and their privacy self-efficacy influence their privacy concern and subsequently mindful sharenting behaviors. Although they have already adopted PMT in the sharenting context, they did not include all theoretical components of PMT, thus falling short of examining the comprehensive relationship between threat-coping appraisals and privacy protection behaviors.

**Figure 1:**
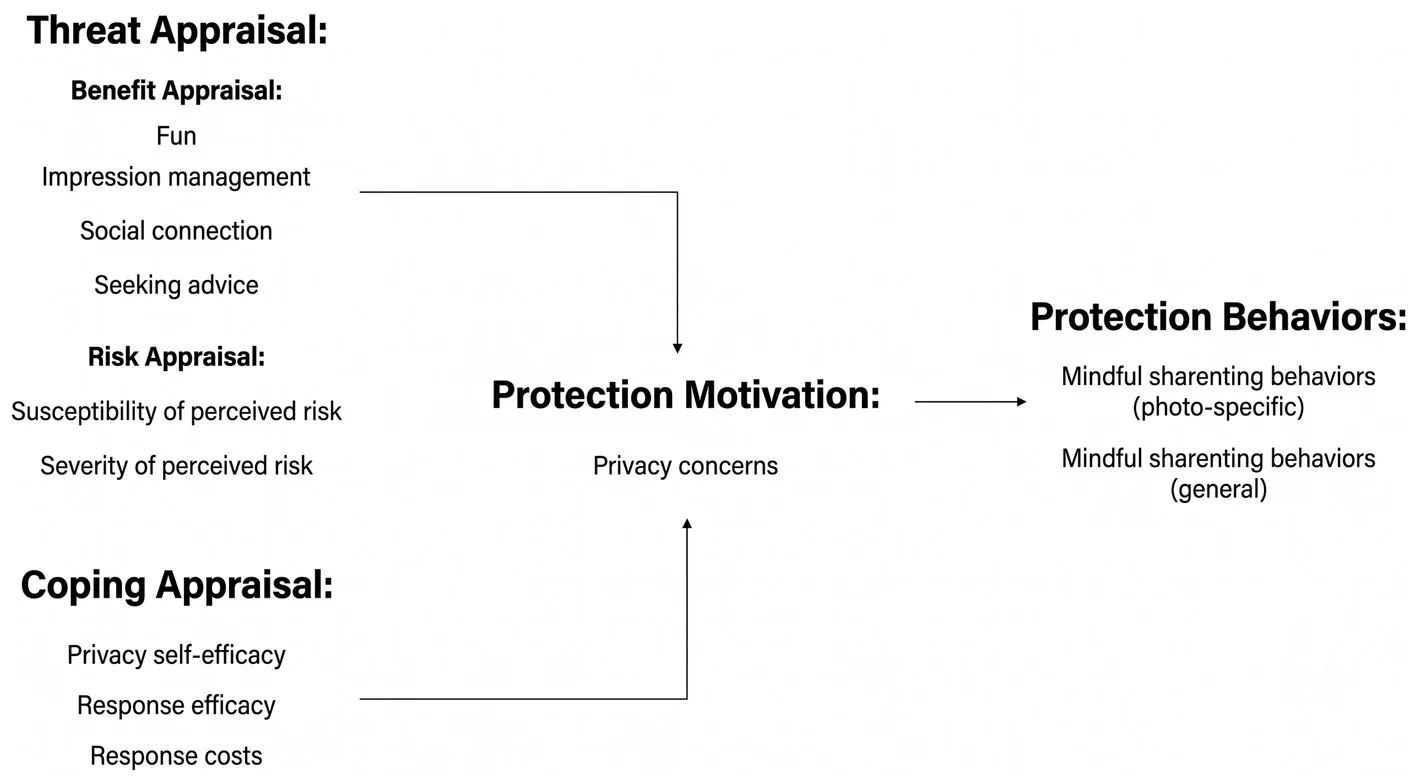
Proposed conceptual model of protection motivation theory predicting mindful sharenting.

To fill this void, our study applies the whole theory of protection motivation to mindful sharenting research and analyzes how each theoretical component of threat (benefits, risk susceptibility, and risk severity) and coping (self-efficacy, response efficacy, and response costs) appraisals influence privacy concerns and the mindful sharenting behaviors (see Figure 1). Given that mindful sharenting as a privacy management behavior involves parents’ perception of potential threats and coping strategies, PMT is theoretically relevant.

This study also investigates the moderating role of a child’s age in examining psychological factors that affect parents’ mindful sharenting strategies. Extant studies indicate that a child’s age is considered important in explaining parental decisions on sharenting (Amon et al., 2022; Berg et al., 2024; Garmendia et al., 2022; Walrave et al., 2022). In the survey study with Spanish school children (9–17 years old), Garmendia et al. (2022) uncovered that pre-adolescents 9–12 years old have started to show some discomfort resulting from sharenting and ask parents to remove some content about them posted online. The observation that children at such a young age have just begun to recognize the possible negative consequences of sharenting may suggest that parents realize the need to carefully balance between the risks and benefits of this practice. However, when children are very young (e.g., 0–8 years old) and unable to form and express opinions on their parents’ sharenting, there is a possibility that parents might overlook their children’s perspectives (Walrave et al., 2022), leading to an underestimation of potential future conflicts with children. Thus, parents’ engagement in mindful sharenting practices may vary in relation to the age of their children (Amon et al., 2022; Walrave et al., 2022). Specifically, threat and coping appraisals are expected to have differential impacts on parents’ privacy concerns and in turn their mindful sharenting depending on children’s age.

Altogether, this study seeks to fulfill the following three primary objectives: (1) determining how threat and coping appraisals within PMT account for parental privacy concerns; (2) assessing whether privacy concerns, acting as protection motivation, lead to risk-reducing mindful sharenting; and (3) investigating the way in which a child’s age influences the relationships between threat-coping appraisals, privacy concerns, and mindful sharenting behaviors. The findings of this study offer theoretical and practical insights into privacy protective measures. This study is the first to fully apply all six theoretical components of PMT to mindful sharenting. By integrating a comprehensive array of PMT’s theoretical elements, the findings advance our knowledge of the underlying processes through which threat and coping appraisals are associated with privacy concerns and mindful sharenting. From a practical standpoint, understanding why parents do (or do not) engage in mindful sharenting helps educators and policy makers develop effective sharenting-related interventions (Williams-Ceci et al., 2021). Such initiatives can heighten risk awareness and promote safer sharenting practices, which mitigate the potential privacy and safety risks stemming from sharenting behaviors.

## Literature review

### Mindful sharenting as privacy management

Sharenting refers to the behavior in which parents share their children’s personal information with the public (Peng, 2024). As social media becomes integral to parents’ daily life, sharenting has increased rapidly and gained significant academic attention (Brosch, 2016; Cataldo et al., 2022). According to a Parents’ Social Media Habit report (2021), 77% parents in the U.S. shared stories, videos, or images of their children or step-children on social media. In today’s social media age, sharenting is considered as a social and cultural norm. Parents who refrain from posting any information about their children online are considered to be deviants of the norm (Siibak and Traks, 2019).

Discussion on sharenting involves a complex trade-off between its benefits and consequences (Peng, 2024). For parents, sharing online fosters connections with peers navigating similar stages of child-rearing, creating a vital network of mutual support (Livingstone and Blum-Ross, 2017). The social capital built through sharenting could help parents adjust to parenthood. Furthermore, posting children’s updates allows distant family and friends to stay connected to a child’s well-being, and sharing children’s accomplishments can validate parental competence (Kumar and Schoenebeck, 2015; Say et al., 2025). Additionally, the digital recording feature of social media serves as a modern-day scrapbook for parents, capturing their children’s growth milestones and precious moments (Livingstone and Blum-Ross, 2017).

Although sharenting offers multiple benefits to parents, what it concerns most to the public and scholars are the harms and risks it induces to children. When parents post children related posts, the availability and accessibility of these personal data create opportunities for cybercrimes and social harms, including cyberbullying, cybergrooming, fraud, identity theft, and the production or circulation of child sexual abuse material (Barnes and Potter, 2021; Williams-Ceci et al., 2021). Children’s images and information can be misappropriated for “digital kidnapping” (Esfandiari and Yao, 2022) or misused for commercial exploitation (Lavorgna et al., 2023). Sharenting also creates tension between parents and children as parents form their children’s online identities without consent (Steinberg, 2016; Walrave et al., 2022). Identities formed by parents may conflict with children’s real identities as they become adults (Amon et al., 2022).

Awareness of these negative consequences of sharenting, coupled with children’s disapproval, has led parents to exercise greater discretion as to what and how they share on social media (Autenrieth, 2018; Walrave et al., 2023). To mitigate potential risks, parents developed new sharenting practices, which can help parents fulfill the needs of putting children’s pictures online while leaving as few identifiable traces as possible. The behavior in which parents still engage in sharenting but employ privacy protective strategies to limit the potential negative effects of sharenting is called mindful sharenting (Walrave et al., 2023).

Mindful sharenting is a form of general individual privacy management, in which parents use privacy protection strategies to regulate the openness and closing of children’s boundaries to others (Petronio, 2002; De Wolf et al., 2014). Therefore, similar to privacy management, mindful sharenting is not just about closing boundaries but also about using privacy protection strategies to optimize parents’ needs by balancing both private and public disclosure. In online media environment, privacy protection strategies refer to behavioral measures users adopt to protect their personal data online, ranging from basic, provider-prompted security practices (e.g., strong passwords, software updates) to proactive, knowledge-intensive preventive actions (e.g., cookie rejection) that users must initiate themselves (Orszaghova and Blank, 2024). Being mindful means that parents are aware of their motives and goals for engaging in sharenting, as well as understand the effect that sharenting can have on their children and themselves (Walrave et al., 2023). Knowledge of sharenting’s impact could come from negative experiences parents had or heard about from their social circles, such as photo theft, fake accounts, or digital kidnapping (Walrave et al., 2023). To successfully implement privacy protection strategies in sharenting, parents need to combine this knowledge with technical privacy skills (Lieberknecht et al., 2026). Lieberknecht et al. (2026) found that both privacy knowledge and privacy technical skills significantly contribute to parents’ privacy self-efficacy, which in turn strongly predicted privacy protection behaviors.

The awareness of negative impact prompts parents to employ a wide range of strategies to mitigate potential risks associated with sharenting (Walrave et al., 2023). Shared content includes diverse information about children, such as photos, videos, activities, or text (Romero-Rodríguez et al., 2022). Visual-centric social media platforms facilitates sharenting by allowing parents to create digital archives of their children through photos (Amon et al., 2022; Briazu et al., 2021; Holiday et al., 2022; Kallioharju et al., 2023; Ranzini et al., 2020). Yet, sharing photos on social media, which often show an identifiable image of children (e.g., face or body), significantly aggravates the potential risks of identity theft, privacy loss, cyberbullying, and digital kidnapping, compared to text-based posts (e.g., Amon et al., 2022; Romero-Rodríguez et al., 2022; Say et al., 2025; Walrave et al., 2023).

In response to children’s vulnerabilities to privacy risks, scholars have prioritized the study of photo-related sharenting (Amon et al., 2022; Ranzini et al., 2020; Romero-Rodríguez et al., 2022) and a series of practices to maintain a level of visual anonymity and conceal children’s identity (Autenrieth, 2018; Walrave et al., 2023). For example, Walrave et al. (2023) explored several photographic tactics used to obscure children’s identity: such as taking photos from behind or a distance, focusing on specific body parts like hands or feet, and digitally altering images with stickers or cropping. In line with existing literature, this study examines photo-related sharenting as a core mindful practice since parents frequently use photographic masking tactics to hide their children’s identifiable features (Autenrieth, 2018; Walrave et al., 2023).

Beyond safeguarding visual identities, scholars have investigated more general privacy protection strategies to regulate the spread of online posts. As self-censorship practices, parents often protect their children’s privacy by restricting photo access to a trusted circle, such as immediate family and close friends. They bypass public feeds in favor of private channels such as closed WhatsApp groups, restricted Instagram accounts, or dedicated sharing applications like Family Album. Parents also use stricter privacy settings to limit the spread of posts on social media (Walrave et al., 2023). Regulating others’ sharing of information about their children is also actively employed (Lieberknecht et al., 2026; Walrave et al., 2023). Parents enforce “privacy boundaries” within their social circle by establishing explicit rules for relatives about what can be shared and intervening if those rules are violated (Walrave et al., 2023). Building upon the literature on sharenting studies, this study categorizes mindful sharenting into two distinct types: specific photo-related protective behaviors and broader general protective behaviors.

Although parents have begun to develop an awareness of the need to protect their children’s privacy, the adoption of mindful sharenting strategies remains relatively uncommon. Walrave et al. (2023) reported that only 9% of parents abstain entirely from posting photos or videos online. Similarly, Tosuntaş and Griffiths (2024) found that fewer than 25% of parents seek their children’s permission before sharing content about them. Furthermore, according to the Security.org 2021 Parents’ Social Media Habits Report, only approximately 42% of 1,000 surveyed parents actively attempt to limit or filter the details they share about their children, primarily due to concerns regarding identity theft.

As a newly proposed concept, the study on mindful sharenting is still in its infancy phase and more empirical evidence is required to help illustrate the decision mechanism of why parents decide to engage in mindful sharenting. To advance the understanding of mindful sharenting, this study employs PMT to empirically investigate the psychological factors that explain parents’ motivations to engage in mindful sharenting. PMT is theoretically relevant in the context of mindful sharenting because mindful sharenting is considered a privacy protective behavior and PMT helps elucidate how threat-coping appraisals explain protection motivation, which in turn influences mindful sharenting.

### Protection motivation theory

This study applies the protection motivation theory (PMT) as a theoretical framework that guides to explore antecedents of how parents engage in sharenting behaviors of safeguarding their children’s privacy. PMT has two cognitive pillars of the threat and coping appraisals activated when individuals face and protect themselves against risks or dangers (Floyd et al., 2000; Rogers, 1975, 1983). Threat appraisal refers to individuals’ assessment of how severe risks are to them (severity) and how vulnerable they are to these risks (susceptibility) when they engage in risky and maladaptive behaviors. Threat appraisal also includes individuals’ assessment of benefits they can receive by engaging in these risky behaviors (benefit appraisal).

Coping appraisal refers to individuals’ evaluation of how confident they are to cope with risks (self-efficacy) and how effective their responses or actions taken are in reducing risks (response efficacy). Coping appraisal also represents individuals’ evaluation of the costs required to take responses or actions to diminish or remove risks (response costs). Individuals’ responses or actions taken to cope with risks can be either adaptive, which protect themselves from risks, or maladaptive, which do not protect themselves from risks. Both threat and coping appraisals activate individuals’ motivation to protect themselves from risks (protection motivation), which in turn leads to their engagement in protective behaviors (Floyd et al., 2000; Rogers, 1975, 1983).

### Privacy concerns

Grounded on PMT, this study tests the full model that takes into account all antecedents of privacy protection motivation and behaviors in the sharenting context. Specifically, we integrate privacy concerns as a mediator in the PMT model. In the original PMT framework, threat and coping appraisals directly predict protection motivation, and protection motivation predicts subsequent behaviors (Floyd et al., 2000; Rogers, 1975, 1983). In privacy research, privacy concern is commonly conceptualized as the final evaluation that directly precedes behavior (Smith et al., 2011). This mediating role is formalized in the “Antecedents - Privacy Concerns – Outcomes” (APCO) framework, which positions privacy concerns as the central link between antecedents and behavioral outcomes. According to the APCO framework, individual factors (e.g., privacy awareness, past experience of privacy violation) can shape one’s privacy concerns, which in turn will lead to behavioral outcomes such as privacy protection behaviors (Smith et al., 2011; Benamati et al., 2017). Empirically, prior studies in sharenting also support the mediating role of privacy concerns. For example, Peng (2024) argued that parents’ privacy self-efficacy enhanced parents’ sensibility to potential risks, therefore increasing their privacy concerns and potentially resulting in more privacy control and management. Lieberknecht et al. (2026) also found that threat appraisals (e.g., perceiving children as vulnerable, having direct experience with privacy violations) first increase parents’ privacy concerns, which in turn significantly predict privacy protective behaviors. We therefore argue that privacy concerns serve as the mediator through which threat and coping appraisals translate into protective action in the mindful sharenting context.

In the social media landscape, scholars explain privacy concerns with two aspects: social and institutional privacy concerns (Raynes-Goldie, 2010). Social privacy concerns are caused by other users’ (mis)use of individuals’ information and institutional privacy concerns are caused by platforms’ (mis)use of their information. Our study considers both types of privacy concerns (Peng, 2024). Regarding threat appraisal in PMT, privacy concerns as protection motivation are explained by benefit–risk appraisals of sharenting behaviors. It is expected that parents would show a greater level of privacy concerns when they perceive less benefits and more risks of sharenting.

### Threat appraisal

#### Benefit appraisal

For the benefit appraisal to explain parents’ privacy concerns, our study examines two types of benefits: intrinsic (fun) and extrinsic (impression management, social connection, and parental advice) (Kallioharju et al., 2023; Livingstone and Blum-Ross, 2017; Peng, 2024). For intrinsic benefits, it has been found that sharenting itself brings parents enjoyment and fun (Peng, 2024) because this activity is intrinsically entertaining, highlighting hedonic gratification (fun). For extrinsic benefits, scholars argue that impression management serves as a driving force contributing to parents’ disclosure behaviors of their children’s private information. It is likely that parents post about their children to make a favorable impression about their parenthood, status, and reputation (Holiday et al., 2022; Kallioharju et al., 2023; Kumar and Schoenebeck, 2015; Ranzini et al., 2020) (impression management). Sharenting has been found to help parents build and maintain social connection with their family or friends (Briazu et al., 2021; Brosch, 2016; Fox and Hoy, 2019; Peng, 2024). Parents keep distant family and friends updated or restore the lost connections with old friends (Ouvrein and Verswijvel, 2019) when they sharent (social connection). Since being parents is a significant life change, sharenting is served as a coping behavior to deal with parenthood challenges and stresses. Thus, engaging in sharenting behaviors provides parents with benefits such as gaining emotional and social support, exchanging information about similar issues, and getting advice about parenting (Briazu et al., 2021; Fox and Hoy, 2019) (seeking advice). Based on these findings, the current study hypothesizes that parents’ benefit appraisal resulting from sharenting is negatively related to their privacy concerns.

*H1*: As parents perceive more benefits from sharenting, they will show a lower level of privacy concerns.

#### Risk appraisal

As part of the threat appraisal, susceptibility and severity of privacy risks are expected to explain parents’ privacy concerns, which trigger their motivation to protect their children’s privacy online. Scholars have uncovered a wide range of negative consequences resulting from sharenting behaviors (Amon et al., 2022; Briazu et al., 2021; Walrave et al., 2023). Sharenting-related risks include: anxiety, distress, identity theft, cyberbullying, misuse of photographs, potential future conflict in the parent–child relationship, disregard of children’s privacy, and so on (Amon et al., 2022; Ioannou et al., 2021). When parents see these privacy threats as a result of parental sharing, they are more likely to feel worry and fear because they lose control of their children’s personal information. It is expected that parents will show a greater level of privacy concerns when they feel these privacy risks would happen to them. In tandem, when parents perceive these privacy threats to be severe to them and their children, it is predicted that this risk perception will trigger a greater level of privacy concerns. Thus, the following hypotheses are proposed:

*H2*: As parents perceive they are more susceptible to privacy risks from sharenting, they will show a greater level of privacy concerns.

*H3*: As parents perceive privacy risks to be more severe to them and their children, they will show a greater level of privacy concerns.

### Coping appraisal

#### Privacy Self-Efficacy

For coping appraisal, our study takes into account three antecedents: privacy self-efficacy, response efficacy, and response costs. Privacy self-efficacy refers to parents’ confidence in their ability to protect their and their children’s personal information (Lieberknecht et al., 2026; Ranzini et al., 2020). Self-efficacy perception has been considered to be one of the most important factors of explaining protective behavior intentions in PMT, particularly when advanced knowledge and skill sets are required (Floyd et al., 2000). Privacy self-efficacy is found to encourage information disclosure behaviors and engage in implementing privacy control rules (Chen and Chen, 2015; Kang and Oh, 2023). It has also been found that individuals’ confidence in their abilities to protect privacy makes them vigilant to negative consequences related to intrusive technologies’ and/or marketers’ privacy intrusion, thus resulting in higher concerns for privacy (LaRose et al., 2005; Rifon et al., 2005). Similarly, privacy self-efficacy leads to privacy protective behaviors through information withdrawal activities (Dienlin and Metzger, 2016) and influences users’ information privacy concerns in social networks (Adhikari and Panda, 2018).

In the sharenting context, Ranzini et al. (2020) predicted that parents with higher privacy self-efficacy were less likely to engage in photo-sharing activities on Instagram but they found insignificant findings. On the other hand, Peng (2024) reported that parents with higher privacy self-efficacy showed a greater level of privacy concerns about their children. Thus, we expect that parents’ confidence in their privacy management skills may lead to a greater level of privacy concerns because they are more cognizant of potential privacy risks. Based on these arguments, this study proposed the following hypothesis:

*H4*: As parents perceive greater confidence in protecting children’s privacy, they will show a higher level of privacy concerns.

#### Response efficacy

PMT suggests that response efficacy leads to a greater level of protection motivation to prevent threats or risks (Rogers, 1983). Response efficacy represents individuals’ belief of whether responses or actions taken to protect privacy are effective in mitigating privacy threats (Boerman et al., 2021; Lee et al., 2008; Rodríguez-Priego et al., 2022). In the study of Internet safety, Lee et al. (2008) uncovered that individuals tend to adopt virus protection measures when they believe that these measures are effective in diminishing viruses’ harm. By extension, Boerman et al. (2021) found that individuals are more likely to use privacy protective measures (e.g., installing ad blocker, declining to accept cookies, deleting browser history, etc.), when these protective measures are perceived as effective. In the sharenting context, when parents perceive their privacy protective measures are effective in safeguarding children’s privacy, they may feel greater privacy concerns (Boerman et al., 2021; Rogers, 1983). It is likely that parents are highly motivated to engage in mindful sharenting only when they believe their privacy protection measures or actions are effective. Following the empirical findings, the current study hypothesizes:

*H5*: As parents perceive a higher level of response efficacy, they will show a greater level of privacy concerns.

#### Response costs

PMT involves an assessment of response costs, which is individuals’ belief of the financial, time, and effort costs needed to take protective responses or action to reduce risks (Marett et al., 2011). Notably, very few studies have examined the role of response costs in explaining privacy protective behaviors in the PMT framework. Scholars argue that greater response costs lower individuals’ motivation to engage in adaptive responses and increase their motivations to engage in maladaptive responses (Floyd et al., 2000; Rogers, 1983). Simply, protective measures which require greater costs do not seem feasible.

In the context of social media sites, Marett et al. (2011) find that users tend to engage in maladaptive responses such as avoidance (e.g., I try not to think about dangers) and hopelessness (e.g., there is nothing I can do to avoid dangers) when they perceive higher response costs (efforts). We speculate that, if parents feel that response costs to carry out protective measures (e.g., efforts, time, skills, and knowledge) are high, they are more likely to be concerned about their and their children’s privacy because these measures are burdensome, difficult, and time-consuming. Parents who may not have these resources may feel fear or worry about how to prevent privacy loss, which leads to greater privacy concerns. Based on these arguments, the following hypothesis is posited:

*H6*: As parents perceive a higher level of response costs, they will show a greater level of privacy concerns.

### Protective behaviors (mindful sharenting)

In the sharenting context, parents feel anxious or concerned because their children’s private information may be accessed, collected, and misused by platforms and other users. These privacy compromises are likely to generate negative consequences, which motivate parents to engage in mindful sharenting (Walrave et al., 2023). Mindful sharenting involves parents’ vigilant strategies or rules they use to protect their and their children’s privacy and uphold their ownership of private information about their children (Walrave et al., 2023). The literature showcases diverse strategies or rules of mindful sharenting that parents adopt, including photo editing (e.g., disguised child, faraway child, digitally processed child), use of child’s initial or pseudonym, post only to close people, stricter privacy settings, asking people not to share, and so on (e.g., Autenrieth, 2018; Locatelli, 2017; Ranzini et al., 2020; Walrave et al., 2022; Walrave et al., 2023). After reviewing the literature on sharenting, we identified a variety of strategies and rules for mindful sharenting and examined two types of mindful sharenting: photo-specific and general protective behaviors. In relation to privacy concerns, we expect that parents with higher levels of privacy concerns will engage in more mindful sharenting behaviors to safeguard children’s privacy. This expectation aligns with prior research findings on the relationship between users’ information privacy concerns and their privacy protection behaviors in social networks (Adhikari and Panda, 2018). Formally, the following hypotheses are posited:

*H7*: As parents show a greater level of privacy concerns, they will adopt mindful sharenting behaviors: (a) photo-specific protective behaviors and (b) general protective behaviors.

**Figure 2:**
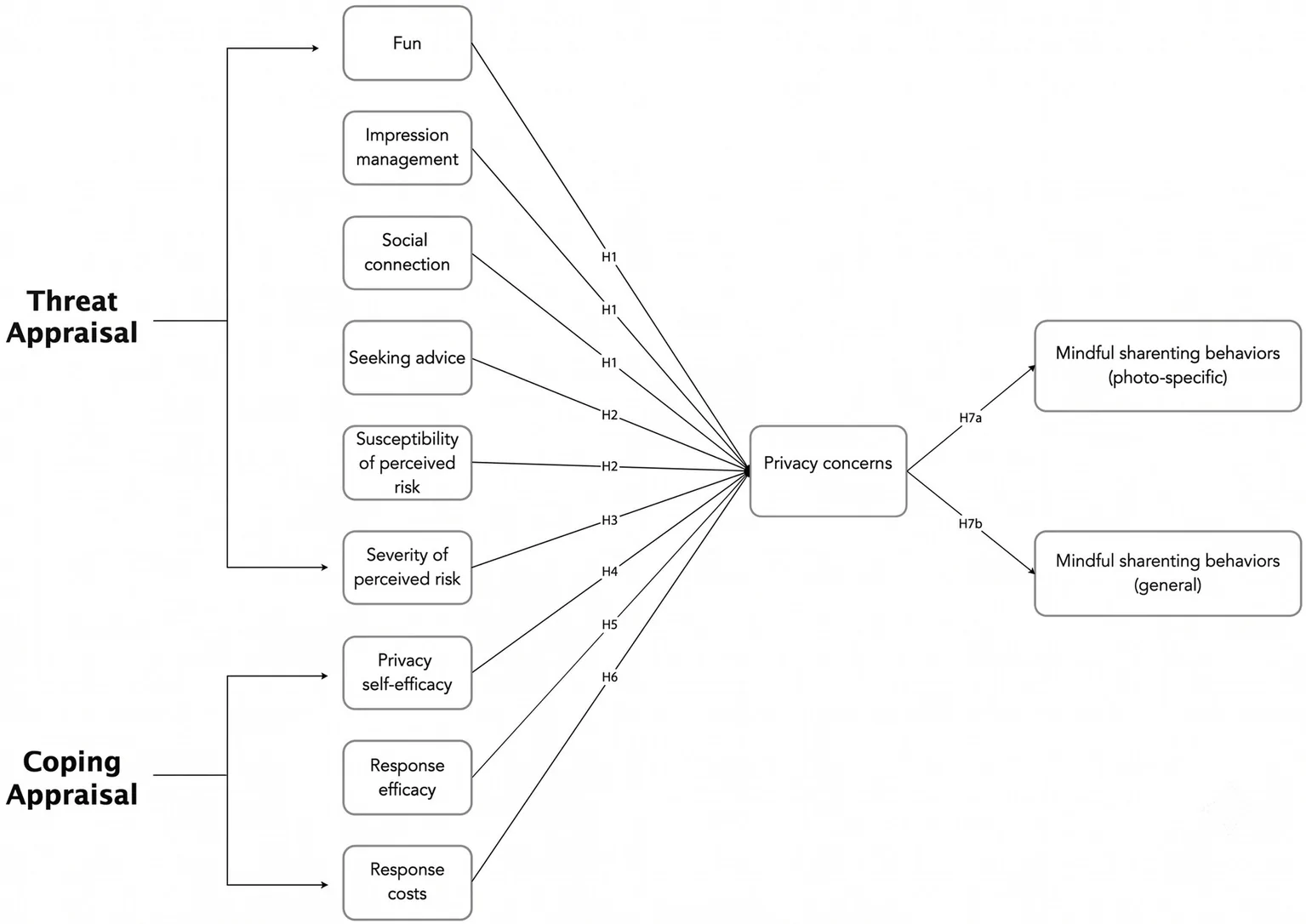
Proposed conceptual model of protection motivation theory with hypothesis. Control variables: parents’ age and parents’ social media use.

All relationships between major constructs are visualized in Figure 2.

### Children’s age as a moderator

Children’s age has long been considered a significant factor influencing the parents’ sharenting decision-making process. There is a general consensus that age 13 is the cutoff at which children begin to realize the potential impact of posted content and ask parents for children’s permission before posting (Amon et al., 2022). From a legal perspective, the Children’s online privacy protection rule (“COPPA”) (2013) sets age 13 as the threshold for online services to collect personal information. Before this age, parents serve as the child’s legal privacy steward and the intermediary between the child and the digital world. When children surpass the 13-year threshold, the parental role shifts from stewardship to surrendering autonomy or sharing sovereignty with the child (Shudra, 2023). This role transition would change the parents’ decision-making mechanism.

From a developmental perspective, the onset of adolescence around age 13 is a crucial period for children’s self-discovery and identity formation (Ouvrein and Verswijvel, 2019; Walrave et al., 2022). Children at this age are concerned about peer impressions and highly value peer validation; they aim to present an “ideal self” online. However, sharented content often conflicts with this desired self-presentation. Levy (2017) found that adolescents feel embarrassed about the content their parents share online. To protect their digital identity and privacy, children negotiate with their parents, requiring them to ask for permission before posting content online (Ouvrein and Verswijvel, 2019; Walrave et al., 2022). If requirements for permission or requests to delete embarrassing content are ignored, children often respond by setting strict boundaries on shareable content to reduce their parents’ sharenting (Ouvrein and Verswijvel, 2021).

Although children’s age is considered a key factor in sharenting decisions, few empirical studies have yet examined how sharenting motivations, attitudes, risk perception, and privacy protective behaviors vary depending on children’s age. Existing literature generally focuses exclusively on children under 13 (Amon et al., 2022; Peng, 2024; Ranzini et al., 2020) or those over 13 (Ouvrein and Verswijvel, 2019, 2021; Walrave et al., 2022). While Williams-Ceci et al. (2021) surveyed parents of children aged 0 to 17, they did not analyze the data by age, though they acknowledged this limitation and called for future research to investigate this specific variable.

While age 13 is the conventional benchmark in sharenting research, scholars increasingly argue that children’s understanding of online privacy begins much earlier (Amon et al., 2022; Garmendia et al., 2022). Amon et al. (2022) argued that parents’ decisions about sharenting might start to change as their children reach pre-adolescence (>age 9) because tweens begin to experience important development changes in social media use, privacy risk perception, and a sense of independence. These important developmental changes are expected to affect parental decisions about sharenting behaviors. Drawing insights from these studies, the present study chooses children’s age nine as our primary cutoff, marking the transition into the tween years. By comparing the sharenting process between parents with younger children (≤age 9) and parents with older children (>age 9), we are able to investigate how a child’s age moderates the impact of threat-coping appraisals on privacy concerns and protective strategies.

Supporting this approach, Berg et al. (2024) urged scholars to examine how sharenting impacts the digital identities of younger children aged 0 to 8. They noted that it is important to study this age group because their digital identities are most often created and formed by parents who post information about their children online without their permission. In tandem, Walrave et al. (2022) contended that parents’ sharenting behaviors vary depending on a child’s age. While younger children lack the developmental capacity to shape and express their views on parents’ sharenting, older children can confront parents and request to remove shared posts (Garmendia et al., 2022; Walrave et al., 2022). Considering that pre-adolescents aged 9 to 12 start to develop critical attitudes toward parents’ sharenting behaviors (Garmendia et al., 2022), it is worth considering children’s age nine - an age on the cusp of the tween years - as a base cutoff when examining the moderating role of children’s age. Building upon these discussions, we propose the following research question:

RQ1: To what extent does a child’s age influence the impact of threat and coping appraisals on privacy concerns, and how do these concerns subsequently affect parents' mindful sharenting behaviors?

## Methods

### Data collection and sampling

A cross-sectional online survey was conducted to test the proposed hypotheses. Before recruiting participants, we obtained IRB approval from an East Coast liberal arts college. We set demographic targeting for participants residing in the United States who were parents of at least one child under the age of 18. In cases where a participant had more than one child under 18, they were instructed to answer the questions about their youngest child. This instruction was given because parents who have sharented about older children may have grown more cautious or knowledgeable about sharenting due to their awareness of privacy risks or experiences stemming from sharenting. Thus, surveying them about the youngest child allows us to capture clearer, more mature views of sharenting behaviors. We established equal quotas for participants across three different child age phases: 0–5 years old, 6–12 years old, and 13–17 years old. Participants were then asked to specify the exact age of the child they were referring to.

We recruited 675 parents from Connect, an online source of high-quality participants by CloudResearch. We paid participants $3 for completing a survey which was expected to take about 15 ~ 20 min. Participants were required to provide informed consent before starting the survey. To fulfill our research objectives, we need to recruit participants who meet the following criteria: (1) 18 years or older; (2) a parent who has at least one child under the age of 18 years old; and (3) having experience of sharing children’s information or parenting experiences online. In our study, “online” refers to any online platforms such as social media (e.g., Facebook, Instagram, X, etc.), blogs, messaging apps (Messenger, WhatsApp, Telegram, etc.), parenting apps, parenting websites, or online parenting forums.

We asked three attention-check questions to ensure the quality of the collected data. Participants were required to write down the age of the child they were currently answering questions about both at the beginning and in the middle of the survey. Participants who provided different child ages (*N* = 52) were excluded from the dataset to secure the quality of data (*N* = 623). The other two attention-check questions required participants to select specific indicated answers, and participants with incorrect answers (*N* = 14) were also excluded (*N* = 609). As we use parents’ age and social media use as the control variables, participants who did not provide answers for these two questions (*N* = 6) were also excluded from the dataset (*N* = 603). After removing all unqualified participants, a total of 603 participants remained for subsequent statistical analyses. Among them, 57% were females. The majority of participants graduated college (50%), some college/vocational school (24%), and graduate school (19%). The majority of participants (41%) reported that their child was 6–12 years old, followed by participants (38%) whose child was 5 years old or younger, and those whose child was 13–17 years old (21%). Of the participants’ children, 63% were 9 years old or younger, whereas 37% were older than 9 years old. Regarding Internet use, most participants reported spending 3–4 h a day (32%), followed by 1–2 h (26%) and 5–6 h (17%), with fewer spending 7–8 h (10%), more than 8 h (9%), or less than 1 h a day (6%). For social media use, the largest group spent 1–2 h a day (38%), followed by 30 min to 1 h (25%) and 3–4 h (18%), with smaller proportions spending less than 30 min (10%), 5–6 h (6%), or more than 7 h a day (2%). Additional demographic information is presented in Table 1.

**Table 1:** Descriptive statistics of participants (*N* = 603).

Demographics	Count	Percentage
Gender		
Female	346	57.4%
Male	253	42.0%
Non-binary	3	0.5%
Prefer not to say	1	0.2%
Education level		
Have not completed high school	3	0.5%
High school graduate	41	6.8%
Some college/vocational school/technical school	145	24%
College graduate	300	49.8%
Graduate school	113	18.7%
Prefer not to answer	1	0.2%
Race and ethnicity		
Asian	32	5.3%
American Indian or Alaska Native	4	0.7%
Black or African American	96	15.9%
Hispanic	53	8.8%
White	412	68.3%
Other	2	0.3%
Prefer not to say	4	0.7%
Household income		
Less than $20,000	20	3.3%
$20,000 to $39,000	73	12.1%
$40,000 to $59,999	96	15.9%
$60,000 to $79,999	107	17.7%
$80,000 to $99,999	74	12.3%
$100,000 to $149,999	141	23.4%
$150,000 or higher	87	14.4%
Prefer not to say	5	0.8%
Marital status		
Single	75	12.4%
Married	448	74.3%
Divorced	54	9.0%
Separated	6	1.0%
Widowed	4	0.7%
Other	12	2.0%
Prefer not to answer	4	0.7%
Time spent on internet		
Never	0	0%
Less than 30 min a day	8	1.3%
30 min–1 h a day	28	4.6%
1–2 h a day	154	25.5%
3–4 h a day	195	32.3%
5–6 h a day	104	17.2%
7–8 h a day	58	9.6%
More than 8 h a day	56	9.3%
Time spent on social media		
Never	1	0.2%
Less than 30 min a day	60	10.0%
30 min–1 h a day	149	24.7%
1–2 h a day	234	38.8%
3–4 h a day	111	18.4%
5–6 h a day	35	5.8%
7–8 h a day	11	1.8%
More than 8 h a day	2	0.3%

### Measures

#### Independent variables

Three items of susceptibility of perceived risk (𝛼 = 0.88) were selected from Banks et al. (2010) and adjusted to fit within sharenting context. Three items of severity of perceived risk (𝛼 = 0.82) were also adapted from Banks et al. (2010) to be in accordance to sharenting context. Benefit appraisal was measured with two types of rewards: Intrinsic and extrinsic. Intrinsic rewards (fun) (𝛼 = 0.90) were measured by three items adopted from Peng (2024). Extrinsic rewards were estimated with three sub-dimensions measured by three items for each: impression management (𝛼= 0.86) (Marett et al., 2011), social rewards (𝛼 = 0.70) (Walrave et al., 2023), and advice rewards (𝛼 = 0.80) (Walrave et al., 2023). Items of social and advice rewards were developed for this study from Walrave et al.’s (2023) qualitative findings on mindful sharenting.

Privacy self-efficacy was estimated with four items (𝛼 = 0.89) derived from Peng’s (2024) privacy self-efficacy scale in sharenting context with one item dropped. One item was dropped because it not only involves sharing children’s information but also parenting experiences, and the latter did not align with the present study. Response efficacy was estimated with three items (𝛼 = 0.90) adapted from Rodríguez-Priego et al. (2022) response efficacy scale. Response costs (𝛼 = 0.93) were measured by four items. Measures of response costs were adapted from Marett et al.’s (2011) response costs question (e.g., effort). Privacy concerns (𝛼 = 0.95) were measured by eight items adapted from privacy concerns scale in Peng (2024) and De Wolf et al. (2014). Items from De Wolf et al. (2014) were changed to fit the sharenting context.

#### Dependent variables

As privacy protective behaviors, mindful sharenting behaviors were adopted from prior studies on sharenting (e.g., Autenrieth, 2018; Locatelli, 2017; Ranzini et al., 2020; Walrave et al., 2022; Walrave et al., 2023). Based on the literature on sharenting studies, we surveyed diverse strategies and rules of mindful sharenting that parents employ online to protect their children’s privacy and created two types of mindful sharenting scale for this study: specific photo-related (7 items, 𝛼 = 0.87) and general (3 items, 𝛼 = 0.70) protective behaviors. Specific wordings, along with descriptive statistics, are found in Table 2.

**Table 2:** Summary of measurement model statistics (*N* = 603).

Construct and measurement items	Mean	SD	Factor loadings
Intrinsic rewards - Fun (*α* = 0.90)			
1. I find sharing my child’s information online to be enjoyable.	4.846	1.413	0.914
2. The actual process of sharing my child’s information online is pleasant.	4.806	1.390	0.840
3. I have fun when sharing my child’s information online.	4.779	1.423	0.866
Extrinsic rewards - Impression management (*α* = 0.86)			
1. I earn respect from family and friends by sharing content of my child with them.	3.537	1.699	0.809
2. Sharing content of my child with others improves my status within my group of friends.	3.217	1.728	0.842
3. I share content of my child with family and friends to improve my reputation.	2.769	1.705	0.818
Extrinsic rewards - Social connection (*α* = 0.70)			
1. By sharing my child’s information online, I could get connected with family and friends.	4.914	1.555	0.651
2. I stimulate conversations with my family and friends.	5.056	1.401	0.599
3. Sharing information about my child online makes me feel like I am part of one or more groups.	3.886	1.819	0.743
Extrinsic rewards – Seeking advice (*α* = 0.80)			
1. By sharing my child’s information online, I could get advice or information about parenting.	3.997	1.752	0.826
2. I share my parenting problems to get support from other parents who are experiencing the same issues.	3.610	1.904	0.792
3. I give my friends parenting advice.	4.032	1.857	0.667
Privacy self-efficacy (*α* = 0.89)			
1. I feel confident in my ability to protect my child’s information because I know how to use the online platform’s privacy settings.	5.481	1.329	0.862
2. I feel in control of who can view my child’s information online.	5.280	1.500	0.887
3. I am capable of using privacy settings on online platforms to control the information about me and my child that I post online.	5.973	1.074	0.683
4. I feel confident that the information I post online about my child can only be seen by those who I have chosen to share it with.	5.227	1.550	0.874
Response efficacy (*α* = 0.90)			
1. If I used privacy protection measures when sharing my child’s personal information online, I could protect my child from losing her or his privacy.	5.595	1.246	0.880
2. Utilizing privacy protection measures when sharing my child’s personal information online helps me ensure my child’s privacy.	5.670	1.221	0.888
3. If I used privacy protection measures when sharing my child’s personal information online, my child would be less likely to lose his or her privacy.	5.612	1.278	0.846
Response costs (*α* = 0.93)			
1. Measures for protecting my child‘s privacy would require a considerable amount of my effort.	4.269	1.828	0.914
2. Measures for protecting my child’s privacy would require a considerable amount of my time.	3.949	1.863	0.861
3. Measures for protecting my child’s privacy would require a considerable amount of my knowledge.	4.605	1.700	0.854
4. Measures for protecting my child’s privacy would require a considerable amount of my skills.	4.237	1.760	0.896
Privacy concerns (*α* = 0.95)			
1. I am concerned that others misuse the information I post about my child online.	4.527	1.760	0.866
2. I am concerned that others can find personal information about my child online.	4.688	1.748	0.888
3. I am concerned that leaving my child’s personal information public online could threaten his or her privacy.	4.857	1.694	0.828
4. I am concerned about posting my child’s information online because it could be used in ways I did not foresee.	4.876	1.648	0.875
5. I am concerned about others placing embarrassing information about my child online.	4.243	1.861	0.745
6. I am concerned that online platforms are collecting too much personal information about my child.	4.723	1.709	0.874
7. I am concerned that online platforms may keep my child’s personal information in a non-accurate manner.	4.592	1.707	0.871
8. I am concerned that online platforms may use my child’s personal information for marketing purposes (e.g., ads).	4.481	1.755	0.830
Susceptibility of perceived risk (*α* = 0.88)			
1. Information I post about my child online could be made available to unknown individuals without my knowledge.	4.431	1.844	0.845
2. I feel my child and I are vulnerable to misuse of the personal information I post about my child online.	3.695	1.897	0.825
3. It is possible that personal information I share about my child online will be used in a way which I would not approve of.	4.249	1.829	0.861
Severity of perceived risk (*α* = 0.82)			
1. If information I post about my child online were misused, it could be damaging to me and my child.	5.307	1.666	0.811
2. If someone misused the information I post about my child online, there could be serious consequences for me and my child.	5.176	1.703	0.798
3. If information I share about my child online was misused, it would bother me and my child.	5.587	1.475	0.733
Mindful sharenting behaviors: Photo-specific (*α* = 0.87)			
1. Photographing the child from a distance.	3.303	1.539	0.681
2. Photographing the child looking away from the camera.	3.161	1.610	0.726
3. Focusing only on a specific body part of the child such as a hand or foot.	2.226	1.496	0.729
4. Covering the face with an emoticon.	2.143	1.607	0.798
5. Blurring the child’s face.	1.954	1.530	0.768
6. Cutting off recognizable parts from the photo.	2.834	1.822	0.675
7. Referring to the child without a photo of the child.	3.539	1.650	0.526
Mindful sharenting behaviors: General (*α* = 0.70)			
1. I share photos only available to a well-chosen group of people rather than entirely public.	5.589	1.649	0.724
2. I use stricter privacy settings of social media profiles.	5.438	1.730	0.787
3. I ask relatives or friends not to share information about my child on online platforms.	4.015	2.145	0.498

Goodness-of-fit statistics: χ^2^ = 2,252.334, df = 968, *p* < 0.001, CFI = 0.934; NFI = 0.890; IFI = 0.934; RMSEA = 0.047.

### Data analysis

A confirmatory factor analysis was performed on a total of 47 items to test the measurement model. Overall goodness-of-fit statistics showed a good fit of the model to the data: Goodness-of-fit statistics: χ^2^ = 2,252.334, df = 968, *p* < 0.001, CFI = 0.934; NFI = 0.890; IFI = 0.934; RMSEA = 0.047. All items represent the construct that they are supposed to correspond with factor loadings of 0.500 to 0.914 (see Table 2). The average variance extracted (AVE) showed 0.500 or greater for the majority of the constructs, showing convergent validity (Fornell and Larcker, 1981). However, the AVE values of three constructs (social connection, photo-specific and general protective behaviors) approached or fell below 0.500, warranting caution in interpretation. Most AVEs were greater than all maximum shared variances (MSVs) and average shared variances (ASVs), except three constructs (impression management, social connection, seeking advice), presenting discriminant validity (Fornell and Larcker, 1981). However, the MSVs of these three constructs are larger than their AVEs because of the multicollinearity involving social connection, which causes these constructs to share more variance with neighboring constructs than with their own items. In the next paragraph, we report this multicollinearity issue and remove social connection from all subsequent analyses. Composite reliability (CR) ranged from 0.705 to −0.953, indicating construct validity (Hair et al., 2009) (see Table 3).

**Table 3:** Correlations, shared variance, CR, AVE, MSV, and ASV (*N* = 603).

Constructs	FUN	IMP	SOC	SKA	SUS	SEV	SEF	REF	COS	PRV	PHO	GEN
Benefits												
Fun (FUN)	**0.763**	0.258	0.646	0.283	0.039	0.003	0.117	0.175	0.001	0.031	0.009	0.001
Impression management (IMP)	0.508	**0.677**	0.682	0.439	0.000	0.027	0.005	0.009	0.067	0.008	0.090	0.039
Social connection (SOC)	0.804	0.826	**0.445**	0.627	0.001	0.000	0.032	0.122	0.023	0.001	0.033	0.002
Seeking advice (SKA)	0.532	0.663	0.792	**0.585**	0.001	0.000	0.018	0.045	0.060	0.010	0.093	0.008
Susceptibility of perceived risk (SUS)	−0.199	0.010	−0.031	0.023	**0.712**	0.118	0.213	0.119	0.084	0.375	0.048	0.000
Severity of perceived risk (SEV)	−0.055	−0.163	0.006	0.020	0.343	**0.610**	0.000	0.003	0.026	0.173	0.003	0.132
Privacy self-efficacy (SEF)	0.342	0.072	0.178	0.134	−0.461	−0.013	**0.690**	0.510	0.030	0.101	0.000	0.202
Response efficacy (REF)	0.419	0.093	0.349	0.213	−0.345	0.051	0.714	**0.760**	0.018	0.056	0.000	0.126
Response costs (COS)	−0.033	0.259	0.152	0.245	0.289	0.163	−0.174	−0.132	**0.777**	0.172	0.073	0.001
Privacy concerns (PRV)	−0.175	0.087	0.025	0.101	0.612	0.415	−0.318	−0.238	0.415	**0.719**	0.086	0.026
*Mindful sharenting*												
Photo-specific (PHO)	0.095	0.301	0.180	0.304	0.219	0.058	−0.011	−0.022	0.270	0.293	**0.498**	0.027
General (GEN)	0.027	−0.198	−0.049	−0.089	−0.004	0.363	0.449	0.354	−0.037	0.161	0.165	**0.464**
No. of Items	3	3	3	3	3	3	4	3	4	8	7	3
Mean	4.810	3.175	4.619	3.879	4.125	5.357	5.491	5.626	4.265	4.622	2.737	5.014
SD	1.290	1.515	1.265	1.555	1.668	1.389	1.197	1.145	1.631	1.504	1.206	1.452
CR	0.906	0.863	0.705	0.807	0.881	0.824	0.898	0.905	0.933	0.953	0.872	0.715
AVE	0.763	0.677	0.445	0.585	0.712	0.610	0.690	0.760	0.777	0.719	0.498	0.464
MSV	0.646	0.682	0.682	0.627	0.375	0.173	0.510	0.510	0.172	0.375	0.093	0.202
ASV	0.142	0.148	0.197	0.144	0.091	0.044	0.112	0.108	0.050	0.094	0.042	0.051

Correlations – below the diagonal; Squared correlations – above the diagonal; Average Variance Extracted (AVE) - on the diagonal and bolded. Composite Reliability (CR), Maximum Shared Variance (MSV), and Average Shared Variance (ASV).

To assess multicollinearity among the latent variables, we reviewed the values of the Variance Inflation Factors (VIFs). Results indicated severe multicollinearity for the extrinsic benefit of social connection (VIF = 22.14), far exceeding the cutoff of 10 (James et al., 2013). After removing the social connection construct, the VIFs of the rest variables were all below 5. According to the correlation matrix, extrinsic social connection strongly correlated with other three benefits variables (*r* = 0.80–0.83). Therefore, the extrinsic benefit of social connection was dropped from all subsequent structural models.

## Results

### Hypotheses testing

**Figure 3:**
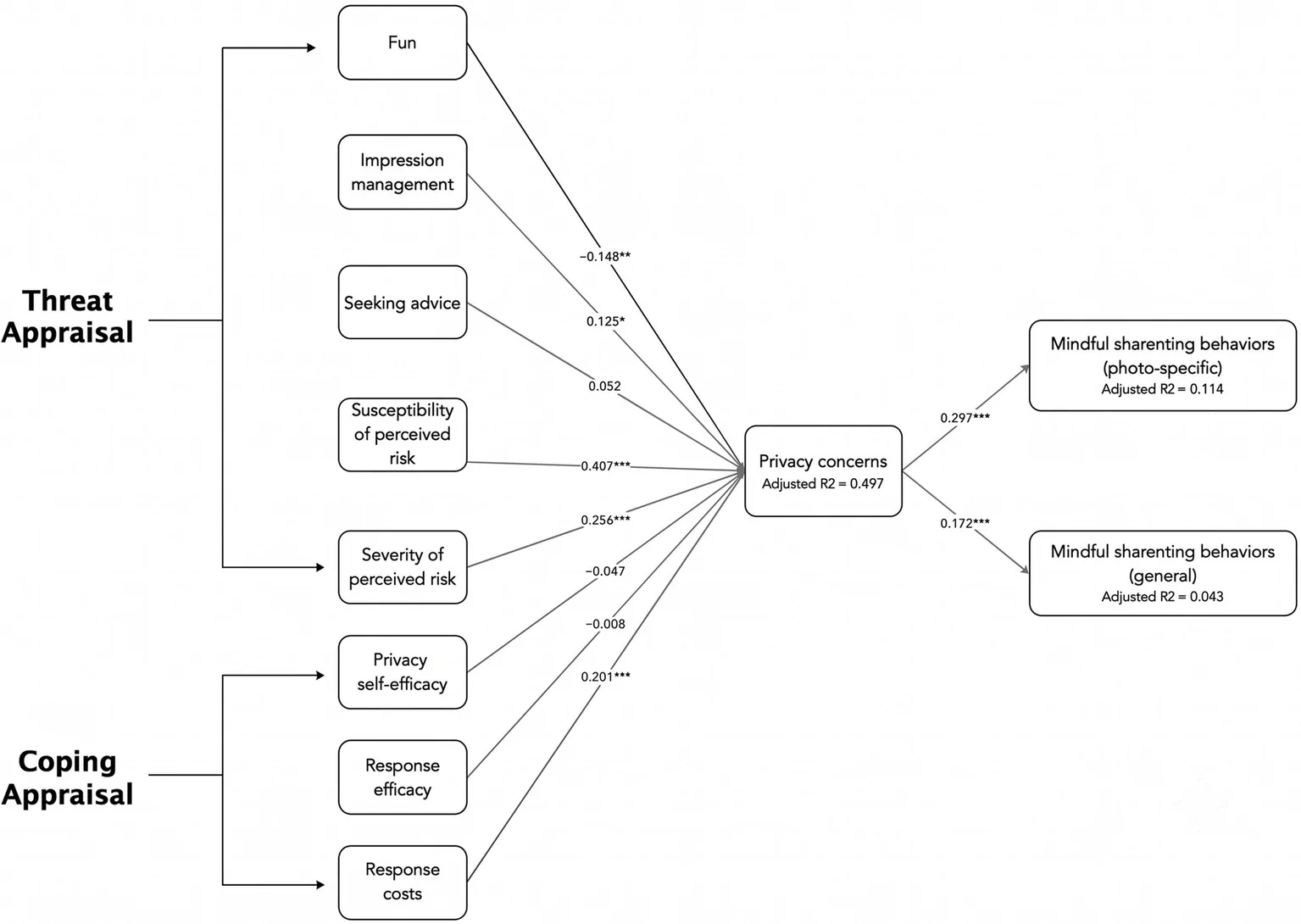
Proposed model with SEM path coefficients. Control variables: parents’ age and parents’ social media use.

A Structural Equation Modeling (SEM) analysis was conducted with RStudio (Version 2024.12.0) to address the hypotheses. It should be noted that parents’ age and social media use were included as covariates in the SEM analysis (*N* = 603). Figure 3 shows the results of the SEM analysis by displaying the structural paths. Table 4 shows the SEM result coefficients of each path. The hypothesized model showed acceptable overall fit to the data, χ^2^ (945) = 2,334.642, *p* < 0.001, CFI = 0.924, TLI = 0.917, RMSEA = 0.049, and SRMR = 0.076. These indices indicate an adequate representation of the observed covariance structure (Kyndt and Onghena, 2014).

**Table 4:** Standardized structural path estimates of all participants (*N* = 603).

Response variables	Predictors	*β*	SE	*p*-value
Privacy concerns	Threat appraisal			
	Fun	−0.148	0.055	0.002
	Impression management	0.125	0.063	0.025
	Seeking advice	0.052	0.060	0.355
	Susceptibility of perceived risk	0.407	0.045	<0.001
	Severity of perceived risk	0.256	0.047	<0.001
	Coping appraisal			
	Privacy self-efficacy	−0.047	0.072	0.390
	Response efficacy	−0.008	0.075	0.885
	Response costs	0.201	0.034	<0.001
Mindful sharenting: Photo-specific	Privacy concerns	0.297	0.031	<0.001
Mindful sharenting: General	Privacy concerns	0.172	0.038	<0.001
	Privacy concerns *Adjusted R*^2^ = 0.497 Mindful sharenting: Photo *Adjusted R*^2^ = 0.114 Mindful sharenting: General *Adjusted R*^2^ = 0.043			

1. Goodness-of-fit statistics: χ^2^ = 2,334.642, df = 945, *p* < 0.001, CFI = 0.924; NFI = 0.879; IFI = 0.925; RMSEA = 0.049, SRMR = 0.076.

2. Control variables: parents’ age and parents’ social media use.

Regarding benefit appraisal, H1 proposed that there would be a negative relationship between perceived benefits and privacy concerns. Results showed that intrinsic fun (𝛽 = − 0.148, *p* = 0.002) was negatively related to privacy concerns, whereas impression management (𝛽 = 0.125, *p* = 0.025) was positively related to privacy concerns. Seeking advice (𝛽 = 0.052, *p* = 0.355) had no significant relationship with privacy concerns. Therefore, H1 was partially supported. For risk appraisal, H2 and H3 hypothesized that susceptibility and severity of perceived risk would be positively related to privacy concerns. As hypothesized, results indicated that susceptibility of perceived risk (𝛽 = 0.407, *p* < 0.001) and severity of perceived risk (𝛽 = 0.256, *p* < 0.001) had a positively significant relationship with privacy concerns. Therefore, H2 and H3 were supported.

Relating to coping appraisal, H4 proposed that privacy self-efficacy would have a positive relationship with privacy concerns. However, results showed that there was no significant relationship between privacy self-efficacy and privacy concerns (𝛽 = −0.047, *p* = 0.390). H5 proposed that response efficacy was positively related to privacy concerns. Like privacy self-efficacy, there was no significant relationship between response efficacy and privacy concerns (𝛽 = −0.008, *p* = 0.885). Therefore, H4 and H5 were rejected. H6 hypothesized that response cost was positively related to privacy concerns. Results showed a positive relationship between response cost and privacy concerns (𝛽= 0.201, *p* < 0.001), thus supporting H6.

H7 hypothesized that privacy concerns would positively relate to both specific photo-related (H7a) and general (H7b) mindful sharenting behaviors. Results indicated that people with higher privacy concerns were more likely to engage in both photo-specific protective behaviors (𝛽 = 0.297, *p* < 0.001) and general protective behaviors (𝛽 = 0.172, *p* < 0.001). Therefore, both H7a and H7b were supported.

### Privacy concerns as the mediator

To test the mediation effect of privacy concerns, we conducted a mediation analysis using 5,000 bootstrap resamples with 95% confidence intervals (Table 5). Effects were considered significant when the confidence interval excluded zero. According to the results, privacy concerns mediated the effects of five of the eight independent variables for both mindful sharenting behaviors, which corroborates the findings of the SEM analysis above. Perceived susceptibility (photo: *β* = 0.090, 95% CI [0.023, 0.105]; general: *β* = 0.11, [0.041, 0.144]), perceived severity (photo: *β* = 0.057, [0.016, 0.085]; general: *β* = 0.070, [0.030, 0.114]), and response cost (photo: *β* = 0.044, [0.010, 0.056]; general: *β* = 0.054, [0.016, 0.077]) exerted positive indirect effects on both photo-specific and general mindful sharenting behaviors. Impression management showed small positive indirect effects on both outcomes (photo: *β* = 0.027, [0.002, 0.053]; general: *β* = 0.034, [0.003, 0.074]), whereas intrinsic-fun benefit showed negative indirect effects (photo: *β* = −0.033, [−0.061, −0.007]; general: *β* = −0.04, [−0.081, −0.011]).

**Table 5:** Indirect effects with privacy concerns as the mediator bootstrap.

Indirect path	B	se	CI-Lower	CI-Upper	*β*	Sig
Mindful sharenting: Photo-specific						
Fun - Photo-specific	−0.026	0.013	−0.061	−0.007	−0.033	*
Impression management - Photo-specific	0.021	0.013	0.002	0.053	0.027	*
Seeking advice - Photo-specific	0.008	0.011	−0.008	0.035	0.011	
Susceptibility- Photo-specific	0.060	0.021	0.023	0.105	0.090	*
Severity - Photo-specific	0.044	0.017	0.016	0.085	0.057	*
Privacy self-efficacy - Photo-specific	−0.010	0.012	−0.040	0.011	−0.011	
Response efficacy - Photo-specific	−0.002	0.013	−0.027	0.024	−0.002	
Response costs - Photo-specific	0.028	0.012	0.010	0.056	0.044	*
Mindful sharenting: General						
Fun - General	−0.037	0.018	−0.081	−0.011	−0.040	*
Impression management - General	0.030	0.018	0.003	0.074	0.034	*
Seeking advice - General	0.012	0.015	−0.012	0.051	0.014	
Susceptibility - General	0.085	0.025	0.041	0.144	0.110	*
Severity - General	0.062	0.020	0.030	0.114	0.070	*
Privacy self-efficacy - General	−0.014	0.017	−0.053	0.015	−0.013	
Response efficacy - General	−0.003	0.017	−0.036	0.034	−0.002	
Response costs - General	0.039	0.015	0.016	0.077	0.054	*

### Age as the moderator

To examine whether children’s age moderated the hypothesized structural relations (RQ1), we conducted a multi-group SEM analysis comparing parents of children aged 9 or younger (*N* = 380) with parents of children older than 9 (*N* = 223), using robust maximum likelihood (MLR) estimation and controlling for parents’ age and social media use. Measurement invariance across the two children’s age groups was tested by comparing a configural model (all parameters freely estimated in each group) with a metric model (factor loadings constrained to equality across groups) (Table 6). Results showed that constraining the factor loadings did not significantly decrease the model fit (Satorra–Bentler scaled Δχ^2^(33) = 27.80, *p* = 0.724), which indicated that the latent constructs were measured equivalently in the two groups, ruling out the possibility that differences in the groups’ structural results merely reflected differences in measurement. We then tested whether the structural paths differed across the two age groups by constraining the 10 focal structural coefficients to equality across groups and comparing this model against the metric-invariance model (Table 7). The equality constraints significantly worsened model fit, Satorra–Bentler scaled Δχ^2^(10) = 23.27, *p* = 0.009, indicating that the structural relations, considered as a set, differed between parents of younger (≤9 years) and older (>9 years) children.

**Table 6:** Measurement invariance.

Model	χ^2^	df	CFI	TLI	RMSEA	SRMR	Δχ^2^ (Δdf)	*p*
Configural	3600.63	1890	0.908	0.900	0.055	0.080	—	—
Metric	3631.43	1923	0.908	0.902	0.054	0.081	27.80 (33)	0.724

**Table 7:** Omnibus test of structural paths across two age groups.

Model	df	χ^2^	AIC	BIC	Δχ^2^	Δdf	*p*
Metric	1923	3631.36	84,477	85,890	—	—	—
Structural	1933	3657.16	84,483	85,852	23.27	10	0.009

Before analyzing a pooled multi-group SEM, separate and single-group SEM analyses were conducted to assess fit indices for each group and compare the findings of each group (Table 8). The analysis of single-group SEMs revealed that the model shows good fit indices for the parent group of younger children. The model fit for the parent of older children appears slightly weak. Thus, these findings should be interpreted with caution, which further requires looking at whether the relationships in the model are strong and theoretically meaningful. Results of single-group SEMs showed that for parents with children aged 9 or younger, susceptibility of perceived risk (*β* = 0.490, *p* < 0.001), severity of perceived risk (*β* = 0.187, *p* < 0.001), and response costs (*β* = 0.251, *p* < 0.001) were significantly related to privacy concerns. For parents with children older than 9, fun (*β* = −0.274, *p* < 0.001) was negatively related to privacy concerns, whereas impression management (*β* = 0.192, *p* = 0.030), susceptibility of perceived risk (*β* = 0.258, *p* < 0.001) and severity of perceived risk (*β* = 0.410, *p* < 0.001) were positively related to privacy concerns. It is notable that privacy concerns were positively related to both photo-specific (*β* = 0.330, *p* < 0.001) and general mindful sharenting (*β* = 0.253, *p* < 0.001) for parents with younger children, but only positively related to photo-specific mindful sharenting (*β* = 0.235, *p* = 0.001) for parents with older children.

**Table 8:** Standardized structural path estimates of parents with children aged 9 or younger and parents with children aged older than 9.

Response variables	Predictors	Children aged 9 or younger (*n* = 380)			Children older than 9 (*n* = 223)		
		*β*	SE	*p*-value	*β*	SE	*p*-value
Privacy concerns	Threat appraisal						
	Fun	−0.096	0.067	0.103	−0.274	0.098	0.001
	Impression management	0.114	0.079	0.111	0.192	0.105	0.032
	Seeking advice	−0.022	0.076	0.755	0.154	0.097	0.088
	Susceptibility of perceived risk	0.490	0.056	<0.001	0.258	0.073	<0.001
	Severity of perceived risk	0.187	0.058	<0.001	0.410	0.082	<0.001
	Coping appraisal						
	Privacy self-efficacy	−0.032	0.095	0.658	−0.041	0.112	0.631
	Response efficacy	0.046	0.097	0.522	−0.149	0.120	0.077
	Response costs	0.251	0.042	<0.001	0.102	0.059	0.092
Mindful sharenting: Photo-specific	Privacy concerns	0.330	0.041	<0.001	0.235	0.046	0.001
Mindful sharenting: General	Privacy concerns	0.253	0.049	<0.001	0.022	0.059	0.782
		Privacy concerns *Adjusted R*^2^ = 0.506 Mindful sharenting: Photo *Adjusted R*^2^ = 0.119 Mindful sharenting: General *Adjusted R*^2^ = 0.072			Privacy concerns *Adjusted R*^2^ = 0.523 Mindful sharenting: Photo *Adjusted R*^2^ = 0.086 Mindful sharenting: General *Adjusted R*^2^ = 0.025		

1. Goodness-of-fit statistics (children aged 9 or younger): χ^2^ = 1,856. 535, df = 945, *p* < 0.001, CFI = 0.922; NFI = 0.854; IFI = 0.923; RMSEA = 0.050, SRMR = 0.080. 2. Goodness-of-fit statistics (children older than 9): χ^2^ = 1,744.080, df = 945, *p* < 0.001, CFI = 0.885; NFI = 0.781; IFI = 0.886; RMSEA = 0.062, SRMR = 0.085. 3. Control variables: parents’ age and parents’ social media use.

To examine whether specific paths differ statistically between two groups, the follow-up Wald tests were performed. Results identified that three paths were significantly different, showing the moderating role of a child’s age (Table 9). The relationship between perceived severity and privacy concerns was stronger among parents of older children (*β* = 0.406, *p* < 0.001) than younger children (*β* = 0.186, *p* = 0.006), Wald χ^2^(1) = 4.57, *p* = 0.033. In contrast, the relationship between privacy concerns and photo-specific protective behaviors was stronger among parents of younger children (*β* = 0.330, *p* < 0.001) than parents of older children (*β* = 0.236, *p* < 0.001), Wald χ^2^(1) = 4.02, *p* = 0.045. The association between privacy concerns and general protective behaviors was significant only among parents of younger children (*β* = 0.252, *p* < 0.001), not among parents of older children (*β* = 0.023, *p* = 0.784), Wald χ^2^(1) = 4.65, *p* = 0.031. Taken together, these differences indicate the moderating role of a child’s age in explaining the relationships between threat-coping appraisals, privacy concerns, and mindful sharenting to safeguard a child’s privacy.

**Table 9:** Wald tests of paths comparison between two age groups.

Path	Children aged 9 or younger		Children older than 9		Wald χ^2^	df	*p* (Wald)
	*β*	*p*	*β*	*p*			
Fun → Privacy concerns	−0.097	0.175	−0.275	<0.001	3.42	1	0.065
Impression management→ Privacy concerns	0.113	0.152	0.194	0.028	0.54	1	0.462
Seeking advice → Privacy concerns	−0.022	0.762	0.153	0.101	2.20	1	0.138
Susceptibility → Privacy concerns	0.487	<0.001	0.266	0.001	3.46	1	0.063
Severity → Privacy concerns	0.186	0.006	0.406	<0.001	4.57	1	0.033*
Privacy self-efficacy → Privacy concerns	−0.033	0.651	−0.037	0.687	0.00	1	0.977
Response efficacy → Privacy concerns	0.046	0.517	−0.151	0.108	2.89	1	0.089
Response costs → Privacy concerns	0.251	<0.001	0.101	0.151	1.99	1	0.159
Privacy concerns → Mindful sharenting: Photo-specific	0.330	<0.001	0.236	<0.001	4.02	1	0.045*
Privacy concerns → Mindful sharenting: General	0.252	<0.001	0.023	0.784	4.65	1	0.031*

## Discussion

Drawing insights from the PMT framework, the present study investigated how psychological factors of threat and coping appraisals activate parents’ motivation to protect their children’s privacy and engage in mindful sharenting behaviors. SEM results indicated that impression management, perceived risk severity, susceptibility, and response costs significantly heightened parents’ privacy concerns, whereas the intrinsic benefit of fun mitigated their privacy concerns. Privacy concerns, in turn, increased the likelihood of engaging in both photo-specific and general mindful sharenting behaviors. Notably, the extrinsic benefit of seeking advice, privacy self-efficacy, and response efficacy were not found to be significant antecedents of parental privacy concerns.

Among the benefit appraisal, the intrinsic benefit of fun and the extrinsic benefit of impression management associated with sharenting influenced parents’ privacy concerns. In line with previous studies, fun was negatively associated with privacy concerns, meaning that when parents find sharing their children’s information enjoyable, their privacy concerns lessen (Arruda Filho et al., 2023). The mediation results show that fun was negatively associated with mindful sharenting through privacy concerns, indicating that parents who perceive sharenting as more fun report lower privacy concerns and are therefore less likely to engage in mindful sharenting (both photo and general). The findings confirmed the PMT proposition that intrinsic rewards, such as the fun derived from sharenting, may attenuate threat appraisal and increase the likelihood of maladaptive behavior (Prentice-Dunn and Rogers, 1986). However, from a mindful sharenting perspective, this finding contradicts Walrave et al. (2023) conclusion that parents try to reap the benefits of sharenting while protecting their children’s privacy at the same time; instead, at least for intrinsic fun, rewards and privacy protection appear to stand in tension rather than coexisting in a reconciled balance.

Interestingly, the SEM and mediation results revealed that impression management was positively associated with privacy concerns, and, in turn, positively associated with mindful sharenting through privacy concerns. This pattern aligns with Kobsa et al. (2012), who argued that a desire for impression management heightens the desire for privacy, because privacy serves as a means of controlling and protecting the image one conveys to others. Applied to sharenting, parents who seek to cultivate an image of a competent parent may become more attentive to what information others can access, precisely in order to safeguard that image; unflattering or unintended information could surface and undermine it, which would therefore intensify their privacy concerns. Because mindful sharenting is itself a form of selective control over what audiences can see and access, heightened privacy concern translates into more careful, protective sharenting.

Seeking advice was associated with neither privacy concerns nor mindful sharenting via privacy concerns. This may be due to parents’ motivations when sharing their children’s information to obtain advice or support from other parents (Peng, 2024). When experiencing the stress and struggles of parenting, parents may want to be authentic and open in their sharing, even though they know the information reveals a vulnerable side of themselves and their children. Parents’ concerns about compromising their own and their children’s privacy may be offset by their motivations to seek advice or support. Consistent with this, Fox and Hoy (2019) found that parents post about their coping experiences with postpartum stress or parenting anxiety to obtain support or affirmation, yet rarely have privacy settings in place to protect their children’s privacy.

Consistent with prior studies, susceptibility and severity of perceived risk were positively associated with privacy concerns and, in turn, positively related to mindful sharenting (Adhikari and Panda, 2018; Boerman et al., 2021). When parents perceive that sharing their children’s information places their children in a vulnerable situation and that the consequences would be severe, their privacy concerns are heightened. The heightened concerns then motivates parents to adopt mindful sharenting to protect their children’s privacy. The significant mediation results of susceptibility and severity of perceived risk on mindful sharenting through privacy concerns confirmed the APCO framework’s positioning of privacy concerns as the central mechanism linking antecedents to behavioral outcomes (Smith et al., 2011). Our findings extend this logic to the sharenting context: parents’ perceptions of how vulnerable their children are, and how severe the potential consequences could be, translate into protective behavior through heightened privacy concerns.

Among the coping appraisal to deal with an ability to cope with risks, our study found that the response costs for privacy protection heightened parents’ privacy concerns; yet privacy self-efficacy and response efficacy did not elevate their privacy concerns. It is notable to discover a positive relationship between response costs and privacy concerns. When parents view response costs (e.g., time, skills, efforts, or knowledge) for safeguarding their children’s privacy as burdensome or onerous, they feel greater fear or worry about their children’s privacy loss. Such sensitivity to high response costs provides valuable insights for understanding which approaches effectively lead to privacy protective behaviors. Lieberknecht et al. (2026) demonstrated a confidence-building approach, finding that privacy literacy, including both privacy knowledge and technical privacy skills, can build parents’ privacy self-efficacy, which in turn translates into actual protective behaviors. In contrast to an efficacy-building approach, our findings shed light on the pivotal role of response costs: when parents perceive technical privacy measures as too burdensome or overly complex to implement, their concerns are heightened, which in turn also translates into protective behaviors.

Unexpectedly, our study did not find positive relationships of parental privacy concerns with privacy self-efficacy and response efficacy. The absence of these significant relationships contradicts previous research on sharenting (Peng, 2024). Indeed, extant studies exhibit mixed findings. Some find that parents with self-efficacy are more cognizant of sharenting risks, thus leading to greater privacy concerns (Adhikari and Panda, 2018; Peng, 2024). Others find that parents with self-efficacy show less privacy concerns because self-efficacy may build a false sense of managing privacy (Lieberknecht et al., 2026). Our findings echo weak association with privacy concerns. Parents with higher privacy self-efficacy may feel more confident in safeguarding their children’s privacy and therefore there are no or less reasons for worrying about their children’s privacy loss. Similar findings were shown for the relationship between response self-efficacy and privacy concerns. Parents who have strong beliefs regarding the effectiveness of privacy protective measures may be less concerned about their children’s privacy, because they think that protective measures are effective in coping with privacy threats (Adhikari and Panda, 2018). Perhaps, biased optimism in parents’ own efficacy and the effectiveness of protective measures may play a role in weakening their concerns about privacy risks. In brief, our findings are in line with prior research that did not find a significant effect of privacy self-efficacy (Boerman et al., 2021; Lieberknecht et al., 2026; Youn, 2009) and response efficacy (Adhikari and Panda, 2018) on privacy concerns. Results on coping appraisals suggest that privacy concerns are not primarily elevated by parents’ self-efficacy or their trust in the efficacy of protective measures. Instead, the perceived costs of implementing protective measures emerge as a key determinant of privacy concerns, which encourage mindful sharenting behaviors.

Aligning with previous research, our study provides further empirical evidence corroborating the positive relationships between privacy concerns and both specific and general mindful sharenting behaviors (Lieberknecht et al., 2026; Walrave et al., 2023). Privacy concerns serve as a major factor of explaining protective behaviors: when parents worry about risks to their children’s privacy, such as cybergrooming, cyberbullying, reputational harm, or identity theft, they may reflect on their sharing behaviors and shift to a protective mode of sharing to mitigate these potential risks (Walrave et al., 2023). Our findings also provide an alternative explanation for previous findings on the privacy paradox phenomenon between privacy concerns and general sharenting behaviors (Briazu et al., 2021; Motevalli et al., 2025; Ranzini et al., 2020). The paradoxical relationship between privacy concerns and sharenting behaviors might represent only part of the full picture. In fact, parents may engage in sharenting and protective measures at the same time. When we observe that parents continue sharenting despite having high privacy concerns, they may have already taken measures to protect their children and control potential harms caused by their sharenting. Therefore, future sharenting and mindful sharenting studies should consider both information disclosure and privacy protective behaviors when including privacy concerns as a mediator to fully understand the underlying mechanism. Supporting this argument, the findings of the mediation analysis demonstrate the role of privacy concerns in explaining the indirect effect of threat-coping appraisals on both mindful sharenting behaviors. In line with the findings of the SEM analysis, privacy concerns serve as a mediator, which accounts for the theoretical mechanism underlying how threat-coping appraisals affect privacy protective behaviors in the sharenting context.

The pooled multigroup SEM analyses revealed the moderating role of a child’s age by comparing two subgroups: parents of younger children (≤9 years) and parents of older children (>9 years). For parents of older children, severity of perceived risk appeared to be a stronger factor of accounting for privacy concerns, compared to parents of younger children. Parents may view negative consequences stemming from sharenting as more severe when children enter pre-adolescence. As older children experience developmental changes, such as growing autonomy, impression management with friends, and the active development of their own digital identities, they begin to challenge parents’ sharenting practices. Parents may experience “privacy turbulence” with their children, leading to familial tension and conflict (Amon et al., 2022; Ouvrein and Verswijvel, 2019; Walrave et al., 2022; Walrave et al., 2023). Beyond interpersonal tension, if information shared about children is misused, it poses severe risks to their safety and privacy, including cyberbullying, identity theft, and digital kidnapping (Walrave et al., 2023).

In addition, the differential impacts of privacy concerns on mindful sharenting behaviors were found between these two groups. Privacy concerns emerged as a stronger trigger for photo-specific protective behaviors for parents of younger children than parents of older children. Regarding general protective behaviors, privacy concerns appeared to be a significant factor for parents of younger children only. It is notable to find that parents of younger children more actively engage in photographic masking tactics for children’s visual anonymity (Autenrieth, 2018; Walrave et al., 2023). Furthermore, parents of younger children actively regulate the spread of shared information by restricting others’ access to information (Walrave et al., 2023). As protection motivation, privacy concerns play a pivotal role in encouraging mindful sharenting for parents of younger children. Parents may feel more responsible for sharenting practices because young children’s digital identities are primarily formed by parental sharing (Donovan, 2020). Altogether, the findings highlight the need for examining the role of a child’s age in sharenting studies.

### Theoretical implications

This study contributes to the sharenting literature by applying the whole theory of protection motivation to privacy protective behaviors. As users’ activities on digital and social media have elevated their vulnerabilities to online risks, PMT has been extensively used in various studies on online security, privacy, and safety (Boerman et al., 2021; Lee et al., 2008; Marett et al., 2011; Rodríguez-Priego et al., 2022; Youn, 2005, 2009). By extension, PMT is theoretically applicable for sharenting studies because mindful sharenting is a protective behavior, which counteracts potential risks such as identity theft, digital kidnapping, cyberbullying, and friction with children. It elucidates how threat and coping appraisals drive protection motivation, which in turn influences mindful sharenting. Scholars have just begun using PMT as a theoretical foundation in the mindful sharenting context (Lieberknecht et al., 2026); yet, comprehensive applications of PMT to mindful sharenting remain scarce. To our best knowledge, this study is the first to investigate all elements of the PMT framework to explore the underlying mechanisms behind how parents engage in mindful sharenting to protect their children against privacy-related risks. Our results provide evidence for the theoretical utility of PMT, as four of its six factors significantly impact protection motivation: perceived risk susceptibility, perceived risk severity, perceived benefits, and response costs.

This study contributes to the PMT literature by including privacy concerns as a mediator in the theoretical model. In both the original PMT framework and previous studies examining online privacy protection behaviors, threat appraisal and coping appraisal variables were typically used as “direct” predictors of privacy protection intentions or behaviors (Boerman et al., 2021; Orszaghova and Blank, 2024; Tsai et al., 2016). Our proposed model introduces an alternative cognitive processing route for understanding how individuals adopt protective behaviors. Specifically, our findings suggest that threat and coping appraisals may not directly lead to protective intentions or behaviors. Instead, these appraisals first heighten individuals’ privacy concerns, subsequently increasing their likelihood of engaging in protective behaviors.

Our study makes a methodological contribution to the sharenting literature by introducing a scale to measure mindful sharenting behaviors. After reviewing the literature on sharenting studies, privacy protective behaviors were categorized into the two types: (1) photo-specific mindful sharenting to maintain children’s visual anonymity and (2) general mindful sharenting to regulate the spread of online posts. This endeavor can serve as a guidepost for future research aimed at formally developing scales in this field.

Notably, this study adds a newer perspective to the sharenting literature by establishing a new age threshold for examining which factors explain protective motivation and protective behaviors. While prior research, influenced by regulatory frameworks like COPPA, has been using 13 years as the standard cutoff (Peng, 2024; Ranzini et al., 2020), our findings suggest that parents’ privacy perceptions relating to a child’s digital age might occur around 9 years old, when children enter pre-adolescence. As digital natives, children start to develop privacy awareness before age 13, leading to earlier negotiations or conflicts between parents and children regarding online sharing (Garmendia et al., 2022; Livingstone, 2009; Stoilova et al., 2021). This explains why parental perceptions of risk susceptibility, along with perceived response costs, begin to show a stronger association with privacy concerns among parents of younger children (≤9 years). As the emerging literature on children’s digital age argues (Amon et al., 2022; Stoilova et al., 2021), it is speculated that pre-adolescents have already begun to develop an understanding that sharing information online could cause potential risks (Garmendia et al., 2022; Ouvrein and Verswijvel, 2019). Our finding aligns with Amon et al. (2022) argument that parental decision-making regarding mindful sharenting starts to change as their children approach pre-adolescence.

### Practical implications

Our study offers practical implications. First, the findings that susceptibility, severity, and response costs influence mindful sharenting via privacy concerns imply that risk-awareness messaging remains a relevant component of intervention design. Lieberknecht et al. (2026) recommended that approaches that strengthen parents’ self-efficacy should be prioritized over fear-based approaches. However, Say et al. (2025) found that children of parents with low awareness of sharenting risks could be more vulnerable to the risks of shared content being abused for commercial purposes. Given that privacy concerns positively predict both specific and general mindful sharenting behaviors, we propose that both approaches should be adopted in interventions that educate parents about sharenting and online privacy. Capability-building interventions that improve parents’ conceptual and technical knowledge of privacy protection are necessary, while interventions that emphasize the susceptibility of children to potential risks, the severity of negative consequences, and the burden of responding to privacy violations can first increase parents’ awareness and draw their attention to this issue (Beuckels et al., 2025). Therefore, interventions could first emphasize the susceptibility to potential risks and the severity of sharenting to increase parents’ attention and concerns, and then provide skills training to build their self-efficacy and increase privacy literacy. When seeking to increase parents’ privacy concerns, interventions should also avoid triggering parents’ defensiveness or making parents feel judged or criticized, which could undermine the effect of privacy concerns (Beuckels et al., 2025). It should be noted that parents may not always have the necessary resources to upskill their privacy knowledge and technical skills for safeguarding their children. Thus, it is essential for other stakeholders such as regulators, policy-makers, or platforms to get involved to reduce the risks associated with sharenting.

The second implication concerns legal regulations. COPPA prohibits online data collection without verifiable parental consent for children under 13 (Youn, 2009), based on the assumption that children of that age lack the ability to comprehend complex online data collection. However, our findings challenge this traditional age cutoff. As children from Generations Z, Alpha, and Beta have largely grown up online, their digital literacy and privacy literacy may develop much earlier than age 13. The findings of our study provide support for this argument. For parents of younger children, privacy concerns were heightened by their assessment of risk susceptibility and the response costs to implement protective measures, but these relationships appeared weaker for parents of older children. Similar findings appeared for the relationship between privacy concerns and mindful sharenting behaviors, with parents of younger children showing more active mindful sharenting as they are concerned about their children’s privacy. This suggests a change in how parents respond to sharenting risks and response costs for protective measures, as their children reach pre-adolescence (9 to 12). Thus, conversations between parents and children regarding sharenting consent might need to begin as early as age nine. It is also worthwhile to consider that legal regulations that empower children to have a say in their digital presence are extended to include pre-adolescents.

The third implication is related to platform design. If the cutoff age for children to provide consent regarding their digital presence is lowered from age 13 to age nine, platforms should offer simplified and gamified privacy settings alongside educational tutorials that match nine-year-old children’s cognitive level, teaching them about online privacy and technical privacy protection. Platforms should also give children the agency to manage content shared by their parents without the child’s permission. For example, platforms could notify children when a parent posts content that includes them and give them the option to request deletion or restrict access to the post. This holds particularly true for digital images that children can be recognizable or identifiable in online posts. Furthermore, platforms could provide a warning that illustrates potential harm to children’s future safety before parents click post to share their children’s information online to heighten their privacy concerns and promote protective awareness (e.g., Peng, 2024).

### Study limitations and future research directions

The limitations of this study provide opportunities for future research. One limitation stems from sampling. This study collected survey participants from Connect, a crowdsourcing platform operated by CloudResearch. To recruit qualified participants for this study, three screening questions were set up in the Qualtrics questionnaire. One of three screening questions is participants’ prior experiences of sharenting online. Given that our research topic relates to sharenting online, some parents might not participate in this study because this screening question may trigger some concern about their and children’s privacy. This could lead to a non-response bias in our sample in that parents who are less concerned about privacy may take part in the survey.

In relation to sampling, the findings of this study have limited external validity because participants do not represent the US parents under study. Although the sample profiles of the current study show a diverse range of demographics, caution should be taken when interpreting the findings. This study recruited the participants residing in the US only as qualified subjects. This led to the exclusion of non-US residents, resulting in a lack of cross-cultural generalizability within the sample. Recent cross-cultural comparative studies indicate that different cultures may shape diverse norms, motivations, attitudes, and practices regarding sharenting, along with varying legal frameworks to protect minors’ privacy (Lendvai, 2024; Pastor-Rodríguez and Suárez-Álvarez, 2025). For example, parents in Western countries may value parental motivations such as self-expression and peer interaction, whereas parents in Eastern countries may place more weight on social connection and archiving experiences (Motevalli et al., 2025). Future sharenting research should address such cultural differences to better understand how sharenting is perceived and practiced in different contexts (Akay, 2026).

Furthermore, there may be the limitation of self-report bias because of a social desirability issue. Participants may provide socially desirable answers to make a good impression about themselves as parents when answering questions about mindful sharenting behaviors. This study also had cross-sectional data constraints. Given our findings that the effect of parents’ threat-coping appraisals and privacy concerns on mindful sharenting varies depending on a child’s age, it is worthy of conducting a longitudinal study with multiple waves. In this way, we can understand how parents shift their approach to mindful sharenting over time as their children age. It should be noted that this study utilized age nine as a primary cutoff for examining the moderating role of the child’s age. This threshold was based on existing literature rather than legal or regulatory guidelines. To provide a more robust empirical justification for this specific age cutoff, future research should investigate and compare the attitudes and reactions toward sharenting between these two age groups.

As another limitation of this study, the limited explanatory power for general (vs. photo-specific) protective behaviors should be discussed. Parents may care about their children’s visual identities which are recognizable to others because visual images such as photos may provide opportunities for more serious negative consequences. Photographic masking tactics may require more strenuous efforts to hide visual identities. On the other hand, general mindful sharenting behaviors focus on regulating the spread of online posts by restricting privacy boundaries. These protective behaviors can be viewed as general privacy management tactics, which may be easier to implement. In terms of measurement issues, photo-specific protective behaviors showed a higher alpha score with 7 items (0.87), whereas general protective behaviors showed a lower alpha score with 3 items (0.70). Thus, the limited power for general protective behaviors can be attributed to both conceptual and measurement factors.

Drawing on PMT, this study primarily focused on how threat-coping appraisals influence parents’ privacy concerns and protective behaviors to safeguard their child’s privacy. Despite its value of fully applying PMT to sharenting, it would be beneficial to investigate mindful sharenting with different theoretical lenses such as theory of reasoned action (Fishbein, 1979), which sheds light on attitudinal and social components. The subjective norm (perceived social pressure) from important social others may play a critical role explaining mindful sharenting. While sharenting appears to be a personal behavior, it is intricately woven into larger societal fabrics and differentiated by both individual and contextual factors (Tosuntaş and Griffiths, 2024).

Future research would benefit from examining diverse boundary conditions, specifically by investigating individual differences as moderators. Promising variables include experiences of privacy turbulence (Walrave et al., 2022; Walrave et al., 2023), parental digital literacy (Barnes and Potter, 2021), and a long-term orientation toward children’s privacy and well-being (Tosuntaş and Griffiths, 2024). Additionally, studies should consider contextual moderators such as countries, which possess different cultural norms and expectations about sharenting. These environmental factors inevitably shape parents’ decision-making mechanisms regarding sharenting (Tosuntaş and Griffiths, 2024).

## Conclusion

In closing, this study is the first to apply all theoretical components of PMT to the mindful sharenting context, investigating the impact of threat-coping appraisals on privacy concerns and then protective behaviors. Among the threat appraisal, perceived risk severity and susceptibility, along with impression management, appeared to heighten parents’ privacy concerns. The intrinsic fun benefit mitigated parents’ privacy concerns. Among the coping appraisal, response costs associated with protective measures emerged as a significant predictor of privacy concerns. These findings highlight the pivotal role of sharenting risk awareness and resources required for protecting children against privacy risks. Parental privacy concerns as a mediator were found to lead to mindful sharenting practices across the photo-specific and general protective behaviors. Notably, this study uncovered the moderating role of a child’s age in explaining the differential effects of risk perception and privacy concerns on mindful sharenting behaviors between the two age groups. These findings challenge the conventional 13-year-old benchmark for legally safeguarding children’s privacy, while arguing for the need that family conversations about consent-based sharenting should begin as children reach pre-adolescence. Altogether, by using PMT, this study enriches our theoretical knowledge of the psychological drivers behind mindful sharenting and offers practical guidelines for parents, educators, and policy-makers on protecting children’s online privacy.

## Data Availability

The datasets generated and analyzed during the current study are not publicly available due to strict participant privacy protocols and IRB restrictions, which mandate that data access is exclusively limited to the study authors. Consequently, the data cannot be shared or made available to third parties. Any further inquiries can be directed to the corresponding author/s.
